# Structural and Electrically Conductive Properties of Plasma-Enhanced Chemical-Vapor-Deposited High-Resistivity Zn-Doped β-Ga_2_O_3_ Thin Films

**DOI:** 10.3390/mi16080954

**Published:** 2025-08-19

**Authors:** Leonid A. Mochalov, Sergey V. Telegin, Aleksei V. Almaev, Ekaterina A. Slapovskaya, Pavel A. Yunin

**Affiliations:** 1 Department of Physics & Optical Science, The University of North Carolina at Charlotte, Charlotte, NC 28223, USA; mochalovleo@gmail.com; 2 Department of Inorganic Compounds Chemistry, N. I. Lobachevsky State University, 603950 Nizhny Novgorod, Russia; telegin@ichem.unn.ru (S.V.T.); slapovskaya@unn.ru (E.A.S.); yunin@ipmras.ru (P.A.Y.); 3 Laboratory of Metal Oxide Semiconductors, Research and Development Center for Advanced Technologies in Microelectronics, National Research Tomsk State University, 634050 Tomsk, Russia; 4 Fokon LLC, 248035 Kaluga, Russia; 5 Institute for Physics of Microstructures RAS, 603087 Nizhny Novgorod, Russia

**Keywords:** gallium oxide, thin films, PECVD, structural properties, electrically conductive properties

## Abstract

A method was developed for plasma-enhanced chemical vapor deposition of β-Ga_2_O_3_:Zn thin films with the possibility of pre-purifying precursors. The structural and electrically conductive properties of β-Ga_2_O_3_:Zn thin films were studied. Increasing the temperature of the Zn source (*T*_Zn_) to 220 °C led to the formation of Ga_2_O_3_ films with a Zn concentration of 4 at.%, at *T*_Zn_ = 230 °C [Zn] = 6 at.% and at 235 °C. [Zn] = 8 at.% At *T*_Zn_ = 23 °C, the films corresponded to the β-Ga_2_O_3_ phase and were single-crystalline with a surface orientation of (–201). As *T*_Zn_ increased, the polycrystalline structure of β-Ga_2_O_3_ films with a predominant orientation of (111) was formed. The introduction of Zn led to the formation of a more developed microrelief of the surface. Raman spectroscopy showed that a small concentration of impurity atoms tended to replace gallium atoms in the oxide lattice, which was also confirmed by the Hall measurements. The concentration of charge carriers upon the introduction of Zn, which is a deep acceptor, decreased by 2–3 orders of magnitude, which mainly determined the decrease in the films’ resistivity. The resulting thin films were promising for the development of high-resistivity areas of β-Ga_2_O_3_-based devices.

## 1. Introduction

Recently, β-Ga_2_O_3_ has attracted increasing attention due to the newly discovered opportunities for its potential application as a basis for developing new semiconductor elements and various optoelectronic devices [1,2,3], power electronics devices [4,5,6,7], gas sensors [8,9], and transparent conductive oxides (TCOs) [10].

Like other metal oxide semiconductors, there is a need to obtain high-quality, high-purity thin layers of β-Ga_2_O_3_ with variable conductive properties over a wide range and with the ability to accurately specify their thickness, phase, and chemical composition. To deposit high-quality, thin layers of β-Ga_2_O_3_, it is advisable to consider methods such as atomic layer deposition (ALD) [11,12], pulsed laser deposition (PLD) [13,14], ion beam sputtering deposition (IBSD) [15], and various modifications of chemical vapor deposition (CVD) [16]. The first three methods have advantages but do not have a high deposition rate, and they also require expensive equipment and special precursors. Many epitaxial methods do not permit the deposition of continuous films with small thicknesses. Additionally, these methods do not permit the pre-purification of the precursors. The purity of the final deposited films depends on the impurity contents of the precursors. We previously developed a facility for depositing metal oxide semiconductor films via plasma-enhanced chemical vapor deposition (PECVD), which has the capability to pre-purify precursors [17,18,19]. The gaseous gallium was purified via distillation in complex oxygen–hydrogen plasma. The process of purification includes oxidation and reduction stages carried out at low pressure in an inductively coupled non-equilibrium plasma discharge, with the plasma treatment facilitating the intensive removal of the contained carbon and most of the metal impurities. The Ga precursors were at least 8N pure, and using high-purity precursors clearly affects the chemical composition of the final β-Ga_2_O_3_ films. Background impurities, even at levels of 0.01–0.1 ppm, significantly affect electrical and optical properties and, to a lesser extent, structural properties [20]. Additionally, PECVD offers high-precision control over the thickness, phase, and chemical composition of β-Ga_2_O_3_ films [17,18,19,21,22,23,24,25].

We have previously reported the possible deposition of both Ga_2_O_3_ [22,23,24] and ZnO [25] using the same PECVD method. Given the potential of this method, developing a technique to deposit and study the properties of these metal-oxide-doped films is appealing. Doping ZnO films with Ga facilitates a high concentration of donors in the semiconductor [26,27]. Additionally, when the concentration of Ga is 1 at.% or higher, two-phase composite films are formed that combine the properties of two metal oxides, and are commonly referred to as GZO films. GZO has attracted interest as a material for developing TCOs [26] and gas sensors [28,29], and it can be concluded that the material may be useful for creating highly conductive functional areas in Ga_2_O_3_-based power and optical electronics devices. On the other hand, functional Ga_2_O_3_-based power and optical electronics devices require high-resistivity areas that can act as drift and buffer layers, guard rings, sensitive layers, and low-dark-current photodetectors [30,31,32,33]. To create high-resistance areas, it is advisable to use Ga_2_O_3_ doped with Zn, and this material was initially investigated to obtain *p*-type Ga_2_O_3_ [34,35,36,37]. However, despite theoretical predictions [36,38], it was not possible to obtain *p*-type conductivity by doping Ga_2_O_3_ with Zn in practice. It was later shown that Zn acts as a deep acceptor [39], compensating for the small donor levels of defects formed during material growth, which leads to an increase in Ga_2_O_3_ resistivity. It is thus possible to create layers with high and low conductivity in the Ga–Zn–O system using PECVD with the same precursors and tooling. This work focuses on the deposition and investigation of the structural and electrically conductive properties of Ga_2_O_3_ films doped with Zn and deposited via PECVD with pre-purification of the precursors. This is the first time that Ga_2_O_3_:Zn films have been deposited via PECVD with pre-purification of Ga and Zn precursors, with the advantages of this technique presented above. PECVD and its various modifications have proven to be scalable, highly reproducible, and easy to automate and implement, leading to their widespread industrial use for deposition of thin semiconductor films for various applications [40].

Experimental studies have shown that Ga_2_O_3_:Zn films and bulk crystals are of interest for TCOs [34], phosphors [41], photocatalysts [42,43], and gas sensors [44]. In many studies, especially those involving the deposition of Ga_2_O_3_:Zn films, high concentrations of Zn (1–10 at.%) were used. To compare our results with those studies, we deposited and investigated Ga_2_O_3_:Zn thin films containing 4 at.%, 6 at.%, and 8 at.% of Zn.

## 2. Materials and Methods

The PECVD installation used in the experiment made it possible to purify the initial Ga and Zn and deposit them onto the substrate [17]. During the PECVD process, metal sources were heated to high temperatures at which the formation of appropriate vapors is possible. These vapors interacted with O_2_ and Ar plasma during deposition. The inductively coupled non-equilibrium plasma discharge was excited via an external inductor placed on the outer surface of the reactor, a generator with an operating frequency of 40.68 MHz and the maximum power of 500 W, and a matching unit. Commercially available high-purity Ga 6N and Zn 5N were preliminarily purified: Quartz boats with the initial molten Ga and Zn were placed in the high-temperature zone of a tubular reactor. The purifying reactor was evacuated to 1 × 10^−5^ Torr for a few hours to remove traces of O_2_ and H_2_O from the walls. Next, the Ga source was heated to *T*_Ga_ = 850 °C; then, the total flow of the Ar through the plasma–chemical reactor was set 30 mL/min at the total pressure in the system of 1 × 10^−3^ Torr and the plasma discharge was ignited. The adjacent purifying reactor contained a Zn source heated to temperatures of *T*_Zn_ = 220 °C, 230 °C, and 235 °C. The absolute error of the *T*_Ga_ and *T*_Zn_ was 1 °C. Purified Ga and Zn vapors in elemental form were transported by a carrier gas H_2_ stream through a heated quartz line to a cross-shaped mixing device, which was also equipped with an external heater made of high-purity quartz which served as a growth reactor. The reaction of interaction between the elements was initiated by electron impact/electron sticking mechanisms in the plasma discharge, with a mixture of O_2_ and Ar forming the plasma. Solid reaction products were deposited on a c-plane sapphire substrate. The plasma power corresponded to 30 W at film deposition. The temperature of the substrate *T*_s_ was maintained at 350 °C, a value previously found to be optimal for depositing high-quality Ga_2_O_3_ thin films via PECVD [24]. The area of the substrates was 10 mm × 10 mm, and the average film thickness was 300 nm; the deposition time of thin films was 60 min. The experimental PECVD installation and the principles of its operation were discussed in detail in our previous work [18].

To determine the phase composition of thin films, an X-ray diffraction (XRD) analysis was performed using a Bruker D8 Discover diffractometer (Bruker, Billerica, MA, USA) (CuK_α_ λ = 1.5406 Å) in Bragg–Brentano geometry with a LynxEYE position-sensitive linear detector (Bruker, USA). The morphological state of the surface of gallium oxide films was studied using atomic force microscopy (AFM) using a scanning probe microscope SPM-9700 (Shimadzu, Kyoto, Japan) in contact mode and scanning electron microscopy (SEM) JSM IT-300LV SEM (JEOL, Tokyo, Japan) at an accelerating voltage of 20 kV. The SPM-9700 (Shimadzu, Kyoto, Japan) microscope has a horizontal resolution of 1–2 nm and a vertical resolution of 0.1–0.2 nm. Energy-dispersive X-ray (EDX) spectroscopy performed with an X-MaxN 20 energy-dispersive elemental analysis detector (Oxford Instruments, Oxfordshire, UK) was used to determine the elemental composition of thin films. The error in determining the concentration of elements via this method is 0.1 at.%. In addition, Raman spectra of the thin films were measured using an Alpha 300 AR confocal Raman spectroscopy system (WiTec, Ulm, Germany). A solid-state laser with an operating wavelength of 488 nm and an ×100 objective lens (NA = 0.75) was focused on the surface of the samples. An identical lens collected scattered light in the backscattering geometry. A spectral resolution of 1.2 cm^−1^ was achieved by using a diffraction grating with 1800 lines/mm.

Hall measurements at room temperature were performed to determine the conductivity type, concentration, and mobility of charge carrier of the samples. The four-probe method was used to perform the Hall measurements using the Nanometrics HL5500PC equipment (Nanometrics Inc., Ottawa, ON, Canada), which makes it possible to measure the parameters of high-resistive samples with a resistivity of up to 10^7^ Ohm × cm. The permissible relative error limits when measuring direct current or voltage are ±2%, and ±2% when reproducing current or voltage. The permissible relative error limit for reproducing magnetic induction in the working area is ±2%. Before performing the Hall measurements, Ti/Au contacts were deposited on the thin film surfaces using an MSS-3G-2 magnetron sputtering system. Ti was deposited first.

## 3. Results and Discussion

**Figure 1:**
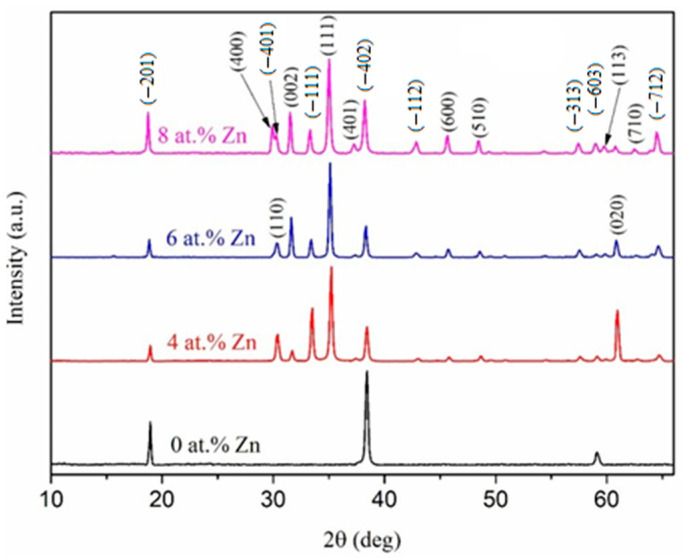
XRD patterns of the PECVD-deposited pure and Zn-doped Ga_2_O_3_ thin films.

On the XRD spectrum of the PECVD-deposited pure gallium oxide thin films (*T*_Zn_ = 23 °C), there are peaks at 2θ = 18.9°, 38.4°, and 59.2° (Figure 1), which can be associated with reflexes (–201), (–402), and (–603) of the β-Ga_2_O_3_ phase (PDF 00-043-10-12), respectively. The single-crystalline β-Ga_2_O_3_ film has the following lattice parameters: *a* = 12.20 Å, *b* = 3.04 Å, *c* = 5.79 Å, and *β* = 103.8°.

With an increase in *T*_Zn_ in addition to the peaks indicated above, multiple peaks appear that are related to reflexes of the β-Ga_2_O_3_ phase on the XRD spectra. In this case, the most intense peak corresponds to the (111) reflex of the β-Ga_2_O_3_ phase. The obtained lattice parameters *a* = 12.23 Å, *b* = 3.05 Å, *c* = 5.80 Å, and *β* = 103.8° are slightly higher in value compared to those of the sample deposited at *T*_Zn_ = 23 °C. Moreover, increasing the *T*_Zn_ results in an increase in the intensity of peaks on the XRD spectrum. Notably, the observed diffraction peaks shift slightly towards smaller 2θ as *T*_Zn_ increases. In addition, an increase in *T*_Zn_ leads to a significant increase in the crystallite sizes (*D*_c_), which were estimated using the Scherrer equation (Table 1). Notably, a high-intensity peak corresponding to the (111) reflex and characteristic changes in lattice parameters were previously observed in β-Ga_2_O_3_ doped with Zn [33,34,42,45,46]. As shown below, an increase in *T*_Zn_ leads to an increase in the Zn concentration [Zn] in β-Ga_2_O_3_ films. Since the ionic radius of Zn^2+^ (0.74 Å) is slightly larger than that of Ga^3+^ (0.62 Å), introducing zinc into β-Ga_2_O_3_, where it substitutes for gallium, increases the lattice parameters of β-Ga_2_O_3_ and shifts the position of the diffraction peaks slightly. An increase in *D*_c_ indicates an increase in the crystallinity of the films with *T*_Zn_.

EDX analysis of the PECVD-deposited pure and Zn-doped Ga_2_O_3_ thin films did not detect the presence of other chemical elements in the films, except Ga, O, and Zn. The concentrations of chemical elements for the studied series of thin films are shown in Table 2. The PECVD-deposited pure Ga_2_O_3_ thin films are characterized via high stoichiometry. An increase in *T*_Zn_ leads to an increase in [Zn]: for films deposited at *T*_Zn_ = 220 °C, [Zn] = 4 at.% is achieved by reducing [Ga] up to 36 at.%. With a further increase in *T*_Zn_ up to 230 °C and 235 °C, an increase in [Zn] is achieved by reducing both [Ga] and [O]. This may be explained by the segregation of the ZnO phase in thin films, characterized by a lower oxygen content. Despite the high concentrations of Zn detected via EDX, no other phases were detected in the films using XRD, which is typical of Zn-doped Ga_2_O_3_ films and crystals, and has been observed previously in Refs. [33,45,46,47]. Optical methods indirectly suggest the formation of ZnO precipitates [48], while the formation of the ZnGa_2_O_4_ spinel phase is possible at Zn concentrations of at least 10 at.% [42,43]. High-temperature annealing also facilitates the formation of the ZnO phase at low Zn concentrations [49].

**Figure 2:**
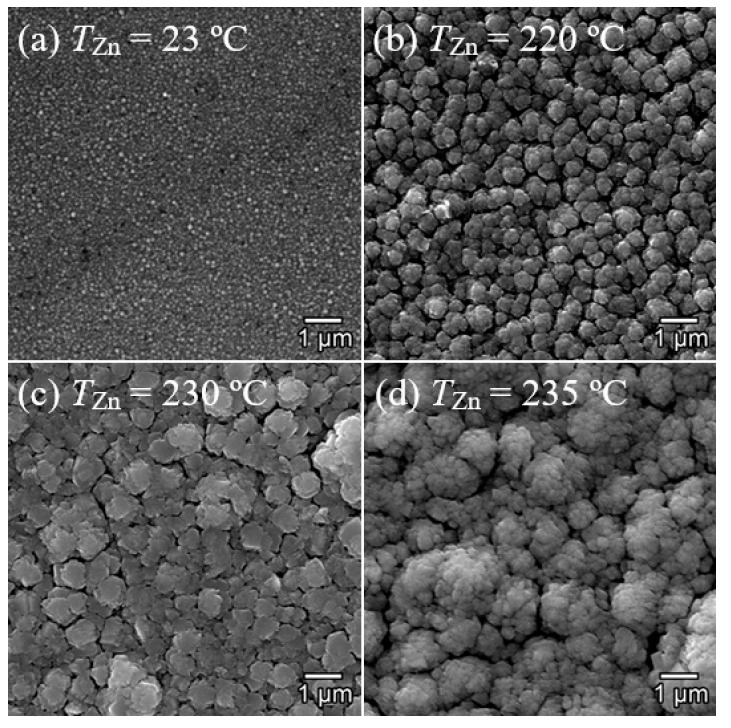
SEM images of the PECVD-deposited pure (**a**) and Zn-doped Ga_2_O_3_ thin film surfaces at *T*_Zn_ = 220 °C (**b**), 230 °C (**c**), and 235 °C (**d**).

The surface of the PECVD-deposited pure Ga_2_O_3_ thin films (Figure 2a) is heteromorphic and is represented by spherical grains with a size of *D*_g_ = 50 nm. An increase in *T*_Zn_ leads to a significant change in the microrelief of the PECVD-deposited Ga_2_O_3_ thin film surfaces. At *T*_Zn_ = 220 °C, large agglomerates up to 500 nm in size are formed, consisting of small grains with *D*_g_ = 50–100 nm. When the *T*_Zn_ increases to 230 °C, the size of agglomerates practically does not change; however, it is not possible to detect small grains. At *T*_Zn_ = 235 °C, the size of the agglomerates reaches 1.5 µm with *D*_g_ up to 300 nm. Notably, for all the studied films, *T*_s_ was fixed at 350 °C, and post-layer annealing was not performed. Even with an increase in *T*_Zn_ by 5 °C, the observed changes in the microrelief of the sample surface are due to an increase in the concentration of Zn in the PECVD-deposited Ga_2_O_3_ thin films (see Table 2 and Figure 2).

**Figure 3:**
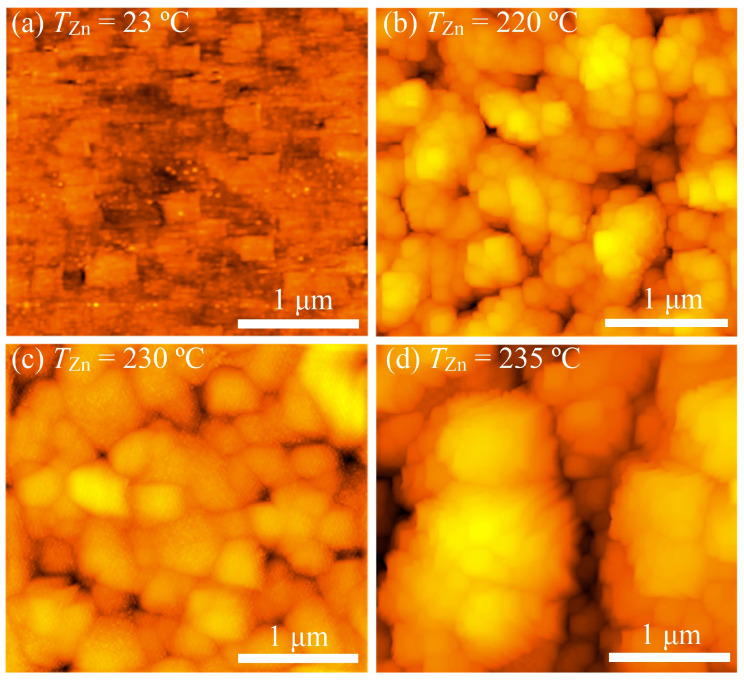
AFM images of the PECVD-deposited pure (**a**) and Zn-doped Ga_2_O_3_ thin film surfaces at *T*_Zn_ = 220 °C (**b**), 230 °C (**c**), and 235 °C (**d**).

AFM images of the PECVD-deposited pure and Zn-doped Ga_2_O_3_ thin film surfaces are shown in Figure 3. The AFM and SEM images of the Ga_2_O_3_ thin films deposited at *T*_Zn_ = 220–235 °C are consistent. However, according to the AFM images, not only small spherical grains but also regular quadrangular crystallites with a size of up to 200 nm appear on the surface of the pure Ga_2_O_3_ thin films, which indicates the monocrystalline structure of the film. The surface roughness parameters of thin films determined using AFM are shown in Table 3, which introduces the following designations: *R*_a_ is the arithmetic average of profile height deviations, *R*_q_ is the root mean square average of profile height deviations, and *R*_z_ is the maximum peak-to-valley height of the profile. The PECVD-deposited pure Ga_2_O_3_ thin films (*T*_Zn_ = 23 °C) are relatively smooth with low roughness parameters. When Zn is introduced into Ga_2_O_3_ thin films (*T*_Zn_ = 220 °C), the roughness parameters increase significantly, which correlates with the SEM results (Figure 2). A further increase in *T*_Zn_ leads to a further increase in *R*_a_, *R*_q_, and *R*_z_. A particularly significant increase in these parameters occurs with an increase in *T*_Zn_ from 230 °C to 235 °C. Similar changes in microrelief and roughness parameters were observed during the doping of β-Ga_2_O_3_ films with Zn [46,49]. Notably, these changes did not always exhibit the same characteristics as the Zn concentration increased.

**Figure 4:**
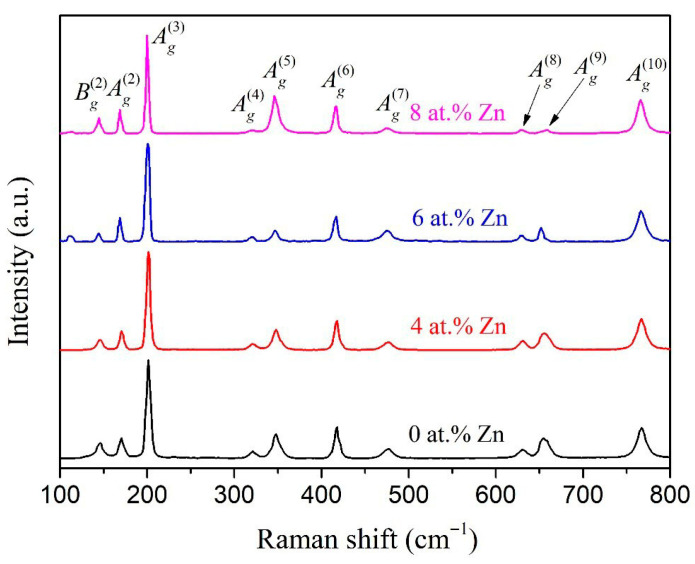
Raman spectra of the PECVD-deposited pure and Zn-doped Ga_2_O_3_ thin films.

Raman spectra of the PECVD-deposited pure and Zn-doped Ga_2_O_3_ thin films are shown in Figure 4. Active phonon modes are closely related to the symmetry of the crystal structure. In the monoclinic structure of the β-Ga_2_O_3_ crystal, there are two [GaO_6_] octahedra connected to two [GaO_4_] tetrahedra forming double chains along the *b*-axis. Each primitive β-Ga_2_O_3_ cell consists of 10 atoms generating 30 phonon modes, 27 of which are optical phonons. These optical modes at the center of the Brillouin zone can be expressed as follows [50]: Γ*^opt^* = 10*A*_g_ + 5*B*_g_ + 4*A*_u_ + 8*B*_u_. Phonon modes with *A*_g_ and *B*_g_ symmetry are Raman-active, while those with *A*_u_ and *B*_u_ symmetry are IR-active.

The presented Raman spectra of β-Ga_2_O_3_ thin films deposited at different *T*_Zn_ are largely similar. The Raman spectra of the samples can be divided into three regions according to the frequency range of the observed vibrational bands [51]. The high-frequency maxima (above 600 cm^−1^) at 631 cm^−1^, 654 cm^−1^, and 767 cm^−1^ correspond to the modes of valence and bending vibrations of GaO_4_ tetrahedra. The peaks at 322 cm^−1^, 348 cm^−1^, 417 cm^−1^, and 477 cm^−1^ in the middle frequency range (300–600 cm^−1^) correspond to the modes of bending vibrations of GaO_6_ octahedra. The main reason for the appearance of bands at 146 cm^−1^and 171 cm^−1^, as well as at 202 cm^−1^ in the low-frequency range (below 300 cm^−1^), is the vibrations and translations of GaO_4_–GaO_6_ chains. The intense peak in the 202 cm^−1^ region (*A_g_*^(3)^ mode) is due to the mode of vibrations of a typical Ga–O chain. Comparing the Raman spectra of β-Ga_2_O_3_ thin films deposited at different *T*_Zn_, it was found that there were no noticeable peak shifts in the spectra. However, the *A_g_*^(3)^ mode in the spectrum of the films is slightly shifted towards lower Raman shift values as *T*_Zn_ increases. In particular, for *T*_Zn_ = 23 °C and 220 °C, the position of this peak is near 202 cm^−1^, and with further increase in *T*_Zn_ up to 230 °C and 235 °C, the peak shifts to 201 cm^−1^ and 200 cm^−1^, respectively. Notably, a similar change in the Raman spectra of β-Ga_2_O_3_ thin films was observed earlier [47,52] when Zn was introduced, which manifested as a shift in the position of *A_g_*^(3)^ to the region of low cm^−1^. This hardly noticeable shift is due to the ionic radius of Zn being close to that of Ga. During the formation of the crystal structure of Ga_2_O_3_ doped with Zn, a small part of gallium in the lattice nodes can be replaced by zinc. Zn ions introduced into the Ga_2_O_3_ lattice break the original Ga–O bond and transform the vibrational or translational modes of GaO_4_–GaO_6_ chains, so it is only when the Zn content increases to a certain extent that the shift becomes more noticeable.

According to the Hall measurements, the pure β-Ga_2_O_3_ thin films (*T*_Zn_ = 23 °C) have *n*-type conductivity, electron concentration *n* = 1.15 × 10^15^ cm^−3^, mobility *µ* = 0.11 cm^2^ × V^−1^ × s^−1^, and resistivity *ρ* = 4.8 × 10^4^ Ohm × cm (Table 4). The relatively high *n* is due to the presence of donor-type defects [53] or background Sn impurity [17,23]. The relatively low *µ* is due to the low thickness of the films and the presence of grain boundaries, which enhances the scattering of charge carriers on the surface and leads to a decrease in *µ* in the thin films [54].

All thin films deposited at *T*_Zn_ = 220–235 °C have *n*-type conductivity. Thin films deposited at *T*_Zn_ = 220 °C are characterized by significantly lower *n* and, consequently, high ρ (Table 4). *µ* increases slightly for films at *T*_Zn_ = 220 °C, and notably, ρ increases mainly due to a decrease in *n*. A decrease in *n* and an increase in the *ρ* of β-Ga_2_O_3_ upon doping with Zn were observed earlier [35,39,55] and result from the Zn impurity being a deep acceptor compensating for the shallow donors. A slight increase in *µ* at *T*_Zn_ = 220 °C and 230 °C (with doping of Zn) is due to an increase in *D*_g_ and other changes in the microrelief of thin films (Figure 2 and Figure 3 and Table 3) [56]. The obtained result is consistent with the Seto model, which was developed for polycrystalline semiconductor films [57]. According to the Seto model, the scattering of charge carriers at grain boundaries is the main mechanism that determines *µ*. An increase in *D*_g_ is observed at *T*_Zn_ = 220 °C and 230 °C, which leads to an increase in *µ*. However, with a further increase in *T*_Zn_ to 235 °C, the microrelief of the films transforms, resulting in the formation of larger agglomerates measuring up to 1.5 µm. These agglomerates consist of smaller grains than those observed at *T*_Zn_ = 220 °C and 230 °C. In this case, it is likely that the smaller grains significantly affect the scattering of charge carriers, leading to a decrease in *µ*.

The results of Raman spectroscopy confirm that the Zn atom replaces the position of the Ga atom. In Refs. [38,58,59,60,61], density functional theory calculations showed that when β-Ga_2_O_3_ is doped with Zn, the substitution of Ga atoms with Zn atoms is an energetically favorable process. Moreover, our results confirm that the changes in the crystal lattice parameters are insignificant. The formation energies of Zn-substituted β-Ga_2_O_3_ vary widely, from 0.9 eV to 5.6 eV, depending on the model and conditions assumed in the calculations. As noted above, the substitution of a Ga atom for a Zn atom creates a deep enough acceptor level (*E*_v_ + (0.7–1.3) eV) that makes it difficult to obtain *p*-type gallium oxide conductivity. The increase in *n* with Zn content (at *T*_Zn_ = 220 °C and 230 °C) can be explained by the possible precipitation of a foreign ZnO phase in the bulk of β-Ga_2_O_3_ films. The content of the ZnO phase is too small to be detected via XRD; however, this semiconductor has a significantly smaller band gap of 3.3 eV [62], which can lead to an increase in the effective concentration of charge carriers. In addition, it is possible to assume the formation of a foreign phase of ZnO doped with Ga during the synthesis and deposition of films. Mutual doping of oxide mixtures was previously established for ITO [63]. On the other hand, Refs. [33,43,64] showed that an increase in Zn leads to an increase in oxygen vacancy concentration, which can exhibit donor-like properties.

The electrically conductive parameters of Zn-doped β-Ga_2_O_3_ thin films have previously been reported by Wang et al. in Ref. [64], where studies of PLD-deposited films at a thickness of 300 nm were carried out. At [Zn] = 3 at.%, 5 at.%, and 7 at.%, *ρ* = 6.97 × 10^6^ Ohm × cm, 2.53 × 10^6^ Ohm × cm, and 2.27 × 10^6^ Ohm × cm, respectively. In addition, the *n* and *µ* values achieved for PECVD- and PLD-deposited Zn-doped β-Ga_2_O_3_ thin films are of a similar order of magnitude. Similar trends are observed in the changes in *n* and *µ*; *µ* values for PLD-deposited films are higher, probably due to the less developed microrelief of the film surface. Notably, the resistivity of β-Ga_2_O_3_ bulk crystals with a Zn concentration of 0.25 at.% was 10^11^–10^14^ Ohm × cm [35].

To develop PECVD technology for β-Ga_2_O_3_ and other ultra-wide bandgap semiconductors, with the possibility of pre-purifying precursors, we plan to conduct detailed studies of their properties to create functional areas for power and sensor electronics devices in the future. The studies will involve analyzing the chemical and elemental composition of films using X-ray photoelectron and secondary ion mass spectroscopy techniques, similarly to the approaches utilized in [65,66].

## 4. Conclusions

A method was developed for plasma-enhanced chemical vapor deposition of β-Ga_2_O_3_:Zn thin films with the possibility of pre-purifying metal precursors. Increasing the temperature of the Zn source (*T*_Zn_) to 220 °C led to the formation of Ga_2_O_3_ thin films with an impurity concentration of 4 at.%, at *T*_Zn_ = 230 °C [Zn] = 6 at.% and at 235 °C [Zn] = 8 at.%. The phase composition of the films, regardless of *T*_Zn_, corresponded to the monoclinic β-Ga_2_O_3_ phase. At *T*_Zn_ = 23 °C, the β-Ga_2_O_3_ films were single-crystalline with a surface orientation of (–201). As *T*_Zn_ increased, a polycrystalline structure of β-Ga_2_O_3_ films with a predominant orientation of (111) was formed. Doping films with Zn led to a slight increase in the lattice parameters of the crystal and a significant increase in the size of the crystallites. The introduction of Zn also led to the formation of a more developed microrelief of the film surface, an increase in roughness parameters by about two orders of magnitude and in grain size by up to 300 nm, and the formation of grain agglomerates with a size of up to 1.5 µm. Raman spectroscopy showed that a small concentration of impurity atoms tended to replace gallium atoms in the oxide lattice, which was also confirmed by the results of the Hall measurements. The concentration of charge carriers upon the introduction of Zn, which is a deep acceptor, decreased by 2–3 orders of magnitude, which mainly determined the decrease in film resistivity. The mobility of charge carriers was determined to a greater extent by the microstructure of the films and weakly depended on [Zn].

## Figures and Tables

**Table 1:** The crystallite sizes of the PECVD-deposited pure and Zn-doped Ga_2_O_3_ thin films.

*T*_Zn_ (°C)	*D*_c_ (nm)
23	30.6
220	32.9
230	34.2
235	36.6

**Table 2:** Elemental composition of the PECVD-deposited pure and Zn-doped Ga_2_O_3_ thin films.

*T*_Zn_ (°C)	Elemental Concentrations (at.%)		
	Ga	O	Zn
23	40	60	0
220	36	60	4
230	35	59	6
235	34	58	8

**Table 3:** Roughness parameters of the PECVD-deposited pure and Zn-doped Ga_2_O_3_ thin film surfaces.

*T*_Zn_ (°C)	*R*_a_ (nm)	*R*_q_ (nm)	*R*_z_ (nm)
23	0.88	1.12	6.47
220	15.17	18.93	55.04
230	23.82	30.51	101.67
235	88.84	106.23	249.55

**Table 4:** Electrically conductive parameters of the PECVD-deposited pure and Zn-doped Ga_2_O_3_ thin films.

*T*_Zn_ (°C)	*ρ* (Ohm × cm)	*n* (cm^−3^)	*µ* (cm^2^ × V^−1^ × s^−1^)
23	4.8 × 10^4^	1 × 10^15^	0.11
220	8.3 × 10^6^	5 × 10^12^	0.13
230	3.4 × 10^6^	8 × 10^12^	0.22
235	2.9 × 10^6^	1 × 10^13^	0.16

## Data Availability

The original contributions presented in this study are included in the article. Further inquiries can be directed to the corresponding author.

## References

[1] Alema F., Hertog B., Ledyaev O., Volovik D., Thoma G., Miller R., Osinsky A., Mukhopadhyay P., Bakhshi S., Ali H. (2017). Solar blind photodetector based on epitaxial zinc doped Ga_2_O_3_ thin film. Phys. Status Solidi A.

[2] Moore A., Rafique S., Llewelyn C., Lamb D., Li L. (2025). A Review of Ga_2_O_3_ heterojunctions for deep-UV photodetection: Current progress, methodologies, and challenges. Adv. Electron. Mater..

[3] Almaev A., Tsymbalov A., Kushnarev B., Nikolaev V., Pechnikov A., Scheglov M., Chikiryaka A. (2024). Self-powered UVC detectors based on α-Ga_2_O_3_ with enchanted speed performance. J. Semicond..

[4] Pearton S.J., Yang J., Cary P.H., Ren F., Kim J., Tadjer M.J., Mastro M.A. (2018). A review of Ga_2_O_3_ materials, processing, and devices. Appl. Phys. Rev..

[5] Higashiwaki M., Jessen G.H. (2018). Guest editorial: The dawn of gallium oxide microelectronics. Appl. Phys. Lett..

[6] Almaev A., Nikolaev V., Yakovlev N., Butenko P., Tsymbalov A., Boiko M., Kopyev V., Krymov V., Kushnarev B., Shapenkov S. (2024). Electroconductive and photoelectric properties of Pt/(100) β-Ga_2_O_3_ Schottky barrier diode based on Czochralski grown crystal. J. Vac. Sci. Technol. A.

[7] Qin Y., Xiao M., Porter M., Ma Y., Spencer J., Du Z., Jacobs A.G., Sasaki K., Wang H., Tadjer M. (2023). 10-kV Ga_2_O_3_ Charge-balance Schottky rectifier operational at 200 °C. IEEE Electron Device Lett..

[8] Zhu J., Xu Z., Ha S., Li D., Zhang K., Zhang H., Feng J. (2022). Gallium oxide for gas sensor applications: A comprehensive review. Materials.

[9] Almaev A.V., Chernikov E.V., Novikov V.V., Kushnarev B.O., Yakovlev N.N., Chuprakova E.V., Oleinik V.L., Lozinskaya A.D., Gogova D.S. (2021). Impact of Cr_2_O_3_ additives on the gas-sensitive properties of β-Ga_2_O_3_ thin films to oxygen, hydrogen, carbon monoxide, and toluene vapors. J. Vac. Sci. Technol. A.

[10] Jeon H.M., Leedy K.D., Look D.C., Chang C.S., Muller D.A., Badescu S.C., Vasilyev V., Brown J.L., Green A.J., Chabak K.D. (2021). Homoepitaxial β-Ga_2_O_3_ transparent conducting oxide with conductivity σ = 2323 S cm^−1^. APL Mater..

[11] Hou C., Liang K., Yang Z., Wang Q., Zhang Y., Chen F. (2025). Thermal atomic layer deposition of Ga_2_O_3_ films using trimethylgallium and H_2_O. J. Cryst. Growth.

[12] Liu X., Wang S., He L., Jia Y., Lu Q., Chen H., Ma F., Hao Y. (2022). Growth characteristics and properties of Ga_2_O_3_ films fabricated by atomic layer deposition technique. J. Mater. Chem. C.

[13] Xu C., Shen L., Liu H., Pan X., Ye Z. (2021). High-quality β-Ga_2_O_3_ films with influence of growth temperature by pulsed laser deposition for solar-blind photodetectors. J. Electron. Mater..

[14] Wu Y., Yan X., Jiang Y., Yao T., Chen C., Ye H. (2024). Microstructure and optical properties of β-Ga_2_O_3_ thin films fabricated by pulsed laser deposition. Thin Solid Films.

[15] Kalanov D., Unutulmazsoy Y., Spemann D., Bauer J., Anders A., Bundesmann C. (2022). Properties of gallium oxide thin films grown by ion beam sputter deposition at room temperature. J. Vac. Sci. Technol. A.

[16] Tak B.R., Kumar S., Kapoor A.K., Wang D., Li X., Sun H., Singh R. (2021). Recent advances in the growth of gallium oxide thin films employing various growth techniques—A review. J. Phys. D Appl. Phys..

[17] Mochalov L., Logunov A., Vorotyntsev V. (2021). Preparation of gallium of the special purity for semiconductors and optoelectronics. Sep. Purif. Technol..

[18] Mochalov L., Nezhdanov A., Strikovskiy A., Gushin M., Chidichimo G., De Filpo G., Mashin A. (2017). Synthesis and properties of As_x_Te_100−x_ films prepared by plasma deposition via elemental As and Te. Opt. Quantum Electron..

[19] Almaev A.V., Yakovlev N.N., Chernikov E.V., Erzakova N.N., Mochalov L.A., Kudryashov M.A., Kudryashova Y.P., Nesov S.N. (2024). Gas sensitivity of PECVD β-Ga_2_O_3_ films with large active surface. Mater. Chem. Phys..

[20] Lee K.C., Weis M. (2024). Charge traps in wide-bandgap semiconductors for power electronics applications. Inorganics.

[21] Kobayashi E., Boccard M., Jeangros Q., Rodkey N., Vresilovic D., Hessler-Wyser A., Döbeli M., Franta D., De Wolf S., Morales-Masis M. (2018). Amorphous gallium oxide grown by low-temperature PECVD. J. Vac. Sci. Technol. A.

[22] Mochalov L.A., Logunov A.A., Kudryashov M.A. (2021). Plasma-chemical deposition of gallium oxide layers by oxidation of gallium in the hydrogen-oxygen mixture. J. Phys. Conf. Ser..

[23] Mochalov L., Logunov A., Gogova D., Zelentsov S., Prokhorov I., Starostin N., Letnianchik A., Vorotyntsev V. (2020). Synthesis of gallium oxide via interaction of gallium with iodide pentoxide in plasma. Opt. Quantum Electron..

[24] Mochalov L., Logunov A., Kudryashov M., Prokhorov I., Sazanova T., Yunin P., Pryakhina V., Vorotuntsev I., Malyshev V., Polyakov A. (2021). Heteroepitaxial growth of Ga_2_O_3_ thin films of various phase composition by oxidation of Ga in hydrogen-oxygen plasmas. ECS J. Solid State Sci. Technol..

[25] Mochalov L., Logunov A., Sazanova T., Kulikov A., Rafailov E., Zelentsov S., Vorotyntsev V. (2020). Zinc oxide nanostructured materials prepared by PECVD as a platform for biosensors. Proceedings of the 2020 22nd International Conference on Transparent Optical Networks (ICTON).

[26] Ponja S.D., Sathasivam S., Parkin I.P., Carmalt C.J. (2020). Highly conductive and transparent gallium doped zinc oxide thin films via chemical vapor deposition. Sci. Rep..

[27] Lin W., Ding K., Lin Z., Zhang J., Huang J., Huang F. (2011). The growth and investigation on Ga-doped ZnO single crystals with high thermal stability and high carrier mobility. Cryst. Eng. Comm..

[28] Taha I., Abdulhamid Z.M., Straubinger R., Emwas A.-H., Polychronopoulou K., Anjum D.H. (2024). Ga-doped ZnO nanoparticles for enhanced CO_2_ gas sensing applications. Sci. Rep..

[29] Ramola R.C., Negi S., Singh R.C., Singh F. (2022). Gas sensing response of ion beam irradiated Ga-doped ZnO thin films. Sci. Rep..

[30] Sheoran H., Kumar V., Singh R. (2022). A comprehensive review on recent developments in ohmic and schottky contacts on Ga_2_O_3_ for device applications. ACS Appl. Electron. Mater..

[31] Li B.T., Zhang X.D., Zhang L., Ma Y.J., Tang W.B., Chen T.W., Hu Y., Zhou X., Bian C.X., Zeng C.H. (2023). A comprehensive review of recent progress on enhancement-mode β-Ga_2_O_3_ FETs: Growth, devices and properties. J. Semicond..

[32] Xu G.W., Wu F.H., Liu Q., Han Z., Hao W.B., Zhou J.B., Zhou X.Z., Yang S., Long S.B. (2023). Vertical β-Ga_2_O_3_ power electronics. J. Semicond..

[33] Guo D., Qin X., Lv M., Shi H., Su Y., Yao G., Wang S., Li C., Li P., Tang W. (2017). Decrease of oxygen vacancy by Zn-doped for improving solar-blind photoelectric performance in β-Ga_2_O_3_ thin films. Electron. Mater. Lett..

[34] Yue W., Yan J.L., Wu J.Y., Zhang L.Y. (2012). Structural and optical properties of Zn-doped β-Ga_2_O_3_ films. J. Semicond..

[35] Jesenovec J., Varley J., Karcher S.E., McCloy J.S. (2021). Electronic and optical properties of Zn-doped β-Ga_2_O_3_ Czochralski single crystals. J. Appl. Phys..

[36] Li C., Yan J.-L., Zhang L.-Y., Zhao G. (2012). Electronic structures and optical properties of Zn-doped β-Ga_2_O_3_ with different doping sites. Chin. Phys. B.

[37] Zhang L., Yan J., Zhang Y., Li T., Ding X. (2012). A comparison of electronic structure and optical properties between N-doped β-Ga_2_O_3_ and N–Zn co-doped β-Ga_2_O_3_. Phys. B Condens. Matter.

[38] Guo Y., Yan H., Song Q., Chen Y., Guo S. (2014). Electronic structure and magnetic interactions in Zn-doped β-Ga_2_O_3_ from first-principles calculations. Comput. Mater. Sci..

[39] Tadjer M.J., Lyons J.L., Nepal N., Freitas J.A., Koehler A.D., Foster G.M. (2019). Editors’ Choice-Review Theory and characterization of doping and defects in β-Ga_2_O_3_. ECS J. Solid State Sci. Technol..

[40] Kabongo G.L., Mothudi B.M., Dhlamini M.S. (2021). Advanced development of sustainable PECVD semitransparent photovoltaics: A review. Front. Mater..

[41] Vasanthi V., Kottaisamy M., Ramakrishnan V. (2019). Near UV excitable warm white light emitting Zn doped γ-Ga_2_O_3_ nanoparticles for phosphor-converted white light emitting diode. Ceram. Int..

[42] Wang X., Shen S., Jin S., Yang J., Li M., Wang X., Han H., Li C. (2013). Effects of Zn^2+^ and Pb^2+^ dopants on the activity of Ga_2_O_3_-based photocatalysts for water splitting. Phys. Chem. Chem. Phys..

[43] Du F., Yang D., Sun Y., Jiao Y., Teng F., Fan H. (2021). Electrospun Zn-doped Ga_2_O_3_ nanofibers and their application in photodegrading rhodamine B dye. Ceram. Int..

[44] Li Y., Trinchi A., Wlodarski W., Galatsis K., Kalantar-zadeh K. (2003). Investigation of the oxygen gas sensing performance of Ga_2_O_3_ thin films with different dopants. Sens. Actuators B Chem..

[45] Su Y., Guo D., Ye J., Zhao H., Wang Z., Wang S., Li P., Tang W. (2019). Deep level acceptors of Zn-Mg divalent ions dopants in β-Ga_2_O_3_ for the difficulty to p-type conductivity. J. Alloys Compd..

[46] Feng Q., Liu J., Yang Y., Pan D., Xing Y., Shi X., Xia X., Liang H. (2016). Catalytic growth and characterization of single crystalline Zn doped p-type β-Ga_2_O_3_ nanowires. J. Alloys Compd..

[47] Jiang J., Zhang J., Song Z. (2020). Influence of Zn doping on the morphology and luminescence of Ga_2_O_3_ low-dimensional nanostructures. J. Lumin..

[48] Remple C., Huso J., Weber M.H., McCloy J.S., McCluskey M.D. (2024). Electron irradiation effects on the optical properties of Hf- and Zn-doped β-Ga_2_O_3_. J. Appl. Phys..

[49] Tao J., Jiang X., Fan A., Hu X., Wang P., Dong Z., Wu Y. (2025). Effect of rapid thermal annealing on the characteristics of micro Zn-Doped Ga_2_O_3_ films by using mixed atomic layer deposition. Nanomaterials.

[50] Dohy D., Lucazeau G., Revcolevschi A. (1982). Raman spectra and valence force field of single-crystalline β-Ga_2_O_3_. J. Solid State Chem..

[51] Kranert C., Sturm C., Schmidt-Grund R., Grundmann M. (2016). Raman tensor elements of β-Ga_2_O_3_. Sci. Rep..

[52] Yan Y., Zhu S., Yang J., Zhang Y., Bai W., Tang X. (2025). Effects of Zn doping on optical properties of polycrystalline β-Ga_2_O_3_. Inorganics.

[53] Kumar M., Kumar V., Singh R. (2017). Diameter tuning of β-Ga_2_O_3_ nanowires using chemical vapor deposition technique. Nanoscale Res. Lett..

[54] Harbeke G. Polycrystalline Semiconductors. Physical properties and applications. Proceedings of the International School of Materials Science and Technology at the Ettore Majorana Centre.

[55] Chikoidze E., Sartel C., Yamano H., Chi Z., Bouchez G., Jomard F., Sallet V., Guillot G., Boukheddaden K., Pérez-Tomás A. (2022). Electrical properties of p-type Zn:Ga_2_O_3_ thin films. J. Vac. Sci. Technol. A.

[56] Orton J.W., Powell M.J. (1980). The Hall effect in polycrystalline and powdered semiconductors. Rep. Prog. Phys..

[57] Seto J.Y.W. (1975). The electrical properties of polycrystalline silicon films. J. Appl. Phys..

[58] Wang Y.-R., Luan S.-Z. (2025). Co-Doping Effects on the Electronic and Optical Properties of β-Ga_2_O_3_: A First-Principles Investigation. Materials.

[59] Tang C., Sun J., Lin N., Jis Z., Mu W., Tao X., Zhao X. (2016). Electronic structure and optical property of metal-doped Ga_2_O_3_: A first principles study. RSC Adv..

[60] Zeng H., Wu M., Cheng M., Lin Q. (2023). Effects of Cu, Zn Doping on the Structural, Electronic, and Optical Properties of α-Ga_2_O_3_: First-Principles Calculations. Materials.

[61] Ma X., Qi N., Zhang M. (2025). First-principles investigation of Zn-doped β-Ga_2_O_3_: Electronic, optoelectronic, and thermodynamic properties. Phys. B Cond. Matter.

[62] Davis K., Yarbrough R., Froeschle M., White J., Rathnayake H. (2019). Band gap engineered zinc oxide nanostructures via a sol-gel synthesis of solvent driven shape-controlled crystal growth. RSC Adv..

[63] Gerasimov G.N., Gromov V.F., Ikim M.I., Ilegbusi O.J., Ozerin S.A., Trakhtenberg L.I. (2020). Structure and gas-sensing properties of SnO_2_-In_2_O_3_ nanocomposites synthesized by impregnation method. Sens. Actuators B Chem..

[64] Wang X., Zhang F., Saito K., Tanaka T., Nishio M., Guo Q. (2014). Electrical properties and emission mechanisms of Zn-doped β-Ga_2_O_3_ films. J. Phys. Chem. Solids..

[65] Dallaev R., Sobola D., Tofel P., Škvarenina Ľ., Sedlák P. (2020). Aluminum Nitride Nanofilms by Atomic Layer Deposition Using Alternative Precursors Hydrazinium Chloride and Triisobutylaluminum. Coatings.

[66] Sharma N., Ilango S., Dash S., Tyagi A.K. (2017). X-ray photoelectron spectroscopy studies on AlN thin films grown by ion beam sputtering in reactive assistance of N^+^/N_2_^+^ ions: Substrate temperature induced compositional variations. Thin Solid Films.

